# The Knowledge About the Impact of Multiple Sclerosis on Pregnancy and Maternity Among Patients with Multiple Sclerosis

**DOI:** 10.3390/jcm15124625

**Published:** 2026-06-14

**Authors:** Ewa Krzystanek, Paweł Gęszka, Mateusz Gawin, Magdalena Fabian, Aleksandra Foryś, Anetta Lasek-Bal

**Affiliations:** 1 Department of Neurology, Faculty of Health Sciences in Katowice, Medical University of Silesia, 40-635 Katowice, Poland; abal@sum.edu.pl; 2 Students’ Scientific Association, Department of Neurology, Faculty of Health Sciences in Katowice, Medical University of Silesia, 40-635 Katowice, Polandmateuszgawin2000@gmail.com (M.G.); aleksandraforys92@gmail.com (A.F.)

**Keywords:** multiple sclerosis, pregnancy, maternity, knowledge, breastfeeding

## Abstract

**Background/Objectives**: Multiple sclerosis (MS) is most frequently diagnosed in young adults of reproductive age. Although current evidence indicates that MS itself does not usually preclude pregnancy or parenthood, patients may still have insufficient knowledge in this area. The aim of this study was to assess knowledge about the relationship of MS and pregnancy, childbirth, breastfeeding, fertility, and parenthood among women and men with MS. **Methods**: This single-center questionnaire-based study included 194 patients with MS: 144 women and 50 men. Participants completed a 12-item paper-and-pen questionnaire assessing general patient-level knowledge. Results were analyzed according to sex and age group: ≤35 years and >35 years. The mean number of correct answers and the proportion of participants reaching the predefined threshold of ≥50% correct answers were calculated. **Results**: Participants aged ≤ 35 years achieved a higher mean number of correct answers than those aged > 35 years: 5.0 versus 3.2, respectively. This difference was also observed among women: 5.9 versus 3.3 correct answers. Among men, no age-related difference was observed: 2.7 versus 2.8 correct answers. The predefined threshold of ≥50% correct answers was reached by 38.5% of participants aged ≤ 35 years and 27.9% of those aged > 35 years. Women had higher percentages of correct answers than men for all questionnaire items. **Conclusions**: Knowledge about MS, pregnancy, childbirth, breastfeeding, fertility, and parenthood was limited in this cohort. Women and younger adults achieved higher knowledge. Education should be proactive, repeated during routine MS care, and addressed to both women and men with MS.

## 1. Introduction

Multiple sclerosis (MS) is a chronic autoimmune disease of the central nervous system characterized by inflammatory processes, demyelination, gliosis, and neuronal loss [1,2]. Pathological changes are typically multifocal and mainly involve the periventricular, proximal cortex, optic nerve, spinal cord, cerebellum, and brainstem [1]. MS affects both genders but is diagnosed approximately three times more often in women than in men [1]. Patients with relapsing-remitting MS usually experience their first symptoms between 20 and 40 years of age [3], although the age at disease onset has increased over the last five decades [4]. Despite substantial advances in pharmacotherapy, MS remains the most common primary neurological cause of disability among young adults in Poland [5].

Because MS often affects women of reproductive age, issues related to pregnancy, childbirth, breastfeeding, and parenthood are clinically important. Studies indicate that 90% of women with MS experience their first symptoms before the age of 50, and approximately 20–33% have children after MS diagnosis [6]. Fertility rates are lower in women with MS than in the general population [7,8]. Concerns related to MS are reported by 30–35% of women as a reason for not planning pregnancy.

Current evidence indicates that MS itself does not usually have a negative effect on pregnancy outcomes or substantially increase obstetric risk [9]. Pregnancy is generally associated with a lower relapse rate, whereas relapse activity may increase in the postpartum period [10]. Pregnancy also had no significant long-term causal effect on the mean EDSS (Expanded Disability Status Scale) at 9 years of follow-up [11]. Therefore, accurate information about pregnancy and MS is important for reproductive counseling and shared decision-making.

The aim of this study was to compare the level of knowledge among women and men with MS regarding the influence of MS on pregnancy, childbirth, breastfeeding, and parenthood. Recent international data suggest that persons with MS may still have insufficient awareness of reproductive health issues related to MS [12]. In a Polish study conducted in 2015, entitled “Motherhood of women with multiple sclerosis (MS)—A conscious decision or unnecessary risk? An Evaluation of the level of knowledge about the disease and pregnancy in women with MS”, Krzystanek et al. assessed 55 women with MS using a questionnaire similar to that used in the present study [13]. That study showed insufficient knowledge and false beliefs regarding MS, pregnancy, and motherhood. However, men were not included in the previous analysis.

The present study extends earlier research by including a larger cohort and both female and male participants. In addition, the use of a similar questionnaire allowed comparison of women’s knowledge over an approximately 10-year period. The results may help assess current awareness among patients with MS and support further discussion on educational needs related to pregnancy, childbirth, breastfeeding, and parenthood in MS.

## 2. Materials and Methods

### 2.1. Study Design and Participants

This was a single-center, questionnaire-based study conducted at the Department of Neurology of the Medical University of Silesia in Katowice-Ochojec, Poland. Data were collected from November 2023 to December 2024. Participants were recruited consecutively from among adult patients with a confirmed diagnosis of multiple sclerosis who attended the neurological outpatient clinic/MS treatment unit. Participation was voluntary and anonymous. Initially, 212 questionnaires were collected. Eighteen questionnaires were excluded because of incomplete data. Therefore, 194 complete questionnaires were included in the final analysis. The final study group consisted of 144 women and 50 men diagnosed with MS.

### 2.2. Questionnaire Development

The study was conducted using a specially designed paper-and-pen questionnaire developed by the authors as a study-specific tool. It was prepared in Polish and administered only to participants fluent in Polish. The first version of the questionnaire was constructed in 2014 on the basis of the medical knowledge available at that time and was used in a previous study concerning knowledge about pregnancy and motherhood among women with MS. For the present study, the questionnaire was reviewed in 2023 and updated according to current knowledge and recommendations regarding MS, pregnancy, childbirth, breastfeeding, and disease-modifying therapy. Some answers were modified, while a high degree of similarity with the original questionnaire was preserved to allow comparison over an approximately 10-year period.

The questionnaire was designed to assess general patient-level knowledge rather than specialist-level knowledge of detailed therapeutic algorithms; therefore, some response options were intentionally simplified.

The questionnaire consisted of three parts: demographic and clinical data; parenthood, pregnancy history, and future reproductive plans; and 12 questions assessing knowledge about MS, pregnancy, childbirth, breastfeeding, fertility, parenthood, and disease-modifying therapy. Correct answers were determined on the basis of the Summary of Product Characteristics for individual disease-modifying therapies, available expert recommendations, and current literature.

### 2.3. Questionnaire Completion Procedure

Patients were invited to participate during routine clinical visits and completed the questionnaire on site at the neurological outpatient clinic/MS treatment unit. Participants completed them independently, without a strict time limit, and could return them at any time during the visit or decide not to return them. If needed, investigators provided only technical or explanatory assistance and did not suggest answers.

### 2.4. Definition of the Knowledge Threshold

For the entire group and for individual subgroups, the average number of correct answers was calculated. In addition, the number and percentage of participants who achieved ≥50% correct answers were determined.

The threshold of ≥50% correct answers was established arbitrarily and was used as a pragmatic descriptive cut-off. It was retained to allow comparison with the previous study performed approximately 10 years earlier and to enable descriptive comparison between subgroups with relatively higher and lower knowledge [13]. This threshold should be interpreted as a predefined study-specific threshold, not as a validated clinical standard of adequate patient knowledge.

### 2.5. Statistical Analysis

Statistical analysis was performed using SYSTAT software, version 12.0, for Windows (Systat Software Inc., San Jose, CA, USA). Continuous and ordinal variables are presented as means and standard deviations, while categorical variables are presented as numbers and percentages. Comparisons of the number of correct answers between groups were performed using the Mann–Whitney U test. Categorical variables, including the proportion of participants reaching the predefined threshold of ≥50% correct answers, were compared using the chi-squared test or Fisher’s exact test, as appropriate. A *p*-value < 0.05 was considered statistically significant.

## 3. Results

### 3.1. Demographic and Clinical Characteristics of the Patients with Multiple Sclerosis

The study included 194 participants: 144 women and 50 men. The mean age was 40.3 years in women and 40.2 years in men. Most participants had relapsing-remitting MS: 89% of women and 80% of men. The mean age at diagnosis was 31.5 years in women and 30.2 years in men. The mean disease duration was 8.8 years and 10 years in men. Detailed demographic and clinical characteristics are presented in Table 1.

### 3.2. Parenthood Status and Future Reproductive Plans

Among the 144 women surveyed, 89 reported having at least one child, and 31 declared a wish to have children in the future, either their first child or another one. One woman was pregnant at the time of questionnaire completion. Among 50 men, 26 reported having children, and 20 declared a wish to have children in the future. Twenty-two women reported that they had tried to conceive after being diagnosed with MS, and 19 women reported pregnancy after MS diagnosis. The disease influenced reproductive decisions in both women and men. MS was reported as a key factor influencing the decision to have children by 22 women and 10 men. Detailed data on parenthood and reproductive plans are presented in Table 2.

### 3.3. Knowledge About MS, Pregnancy, and Parenthood

Participants answered 12 questions assessing their knowledge about the impact of MS on pregnancy, childbirth, breastfeeding, fertility, and parenthood. The questions, possible answers, and correct answers are presented in Table 3. The mean number of correct answers was calculated for the entire group, separately for women and men, and for younger (≤35 years of age) and older participants. These results are summarized in Table 4. Additionally, the number and percentage of participants reaching the predefined threshold of ≥50% correct answers were calculated for each subgroup and are presented in Table 5.

Participants aged ≤ 35 years had a higher mean number of correct answers than those aged > 35 years: 5.0 versus 3.2 correct answers, respectively (*p* < 0.001). This difference was also observed among women. Women aged ≤ 35 years achieved a mean of 5.9 correct answers, compared with 3.3 correct answers among women aged > 35 years (*p* < 0.001). Among men, no significant age-related difference was observed: men aged ≤ 35 years achieved a mean of 2.7 correct answers, whereas men aged > 35 years achieved a mean of 2.8 correct answers (*p* = 0.984). These comparisons were performed using the Mann–Whitney U test.

The proportion of participants reaching the predefined threshold of ≥50% correct answers was 38.5% among participants aged ≤ 35 years and 27.9% among participants aged > 35 years (*p* = 0.135). Among women, this proportion was significantly higher in the younger age group than in the older age group: 48.9% versus 28.9%, respectively (*p* = 0.018). Among men, the corresponding proportions were 11.1% and 25.0%, respectively (*p* = 0.239). These comparisons were performed using the chi-squared test.

These results indicate that younger age was associated with a higher mean knowledge score in the total study group and among women. However, this age-related difference should be interpreted with caution because the proportion of participants reaching the predefined threshold of ≥50% correct answers was not significantly different in the total study population. In men, no significant age-related differences were observed.

Women had a higher percentage of correct answers than men for all 12 questions in both the overall study population and the subgroup of participants aged ≤ 35 years. Detailed data are presented in Table 6 and Table 7.

Women demonstrated the highest level of knowledge for question 6 (“In your opinion, should disease-modifying therapy be discontinued in women with multiple sclerosis before trying to conceive?”) and question 8 (“In your opinion, should disease-modifying therapy be discontinued during pregnancy?”). For both questions, the answers “Yes” and “It depends on the medicine used” were considered correct. These were the only questions in which the percentage of correct answers exceeded the predefined threshold of 50%, reaching 63.9% and 65.3%, respectively. Among men, the corresponding percentages were lower: 48% for question 6 and 38% for question 8.

Even better results were observed among women aged ≤ 35 years: 91.5% answered question 6 correctly, and 83.0% answered question 8 correctly. In these two areas, younger women demonstrated the highest level of knowledge among all analyzed groups.

Women aged ≤ 35 years also reached the predefined threshold of 50% correct answers for question 3 (“In your opinion, how does the postpartum period affect the course of multiple sclerosis?”), with 55.3% of correct answers (“Rather unfavorable—it increases the frequency and severity of relapses”); question 10 (“In your opinion, does multiple sclerosis reduce fertility?”), with 51.1% of correct answers (“No”); and question 11, with 74.5% of correct answers. In the same age group, men achieved lower results: 11.1% for question 3, 22.2% for question 10, and 27.8% for question 11.

In the male subgroup, the percentage of correct answers did not exceed 50% for any question, either in the overall male group or in men aged ≤35 years. These findings suggest limited knowledge among male participants; however, this should be interpreted with caution because of the relatively small size of the male subgroup.

The most challenging questions for the entire group were question 4 (“In your opinion, how does breastfeeding affect the course of the disease?”) and question 7 (“In your opinion, should disease-modifying therapy be discontinued in men with multiple sclerosis before trying to father a child?”). Only 4.6% of women, including 6.4% of women aged ≤ 35 years, and 4.0% of men answered question 4 correctly (“Rather favorable—it decreases the frequency and severity of relapses”). Among men aged ≤ 35 years, no participant answered this question correctly.

It is noteworthy that most respondents were aware of the need to consider treatment discontinuation in women before and during pregnancy. However, a much smaller percentage of respondents knew that treatment discontinuation may also be relevant in men before trying to father a child.

### 3.4. Seeking Information

Women were more likely than men to search for information about MS and pregnancy and childbirth. Overall, 30.5% of women and 14% of men reported that they had searched for such information. The most common sources of information among women were neurologists (21.5%) and websites (20.1%). Among men, websites were the most frequently reported source (8.0%), whereas information from physicians was reported less often (4%). Detailed data are presented in Table 8.

In the 2015 study, approximately one-third of women also reported searching for information on this topic. Interestingly, women who searched for information did not achieve significantly higher knowledge scores than those who did not (*p* = 0.077; Mann–Whitney U test) [13].

## 4. Discussion

The present study assessed knowledge about the impact of MS on pregnancy, childbirth, breastfeeding, fertility, and parenthood among women and men with MS. The main finding was that the overall level of knowledge in this area was limited. Women achieved higher knowledge scores than men, and younger women had higher scores than older women. However, conclusions regarding men should be interpreted with caution because of the relatively small male subgroup, especially among men aged ≤ 35 years. Similarly, the age-related findings should be interpreted cautiously, as the proportion of participants reaching the predefined threshold of ≥50% correct answers was not significantly different in the total study population.

Reproductive decisions in patients with MS are complex and may be influenced by both disease-related and non-disease-related factors. In the present cohort, 89 of 144 women had at least one child, whereas 55 had no children at the time of the survey. Only 31 women declared a wish to have children in the future, and 19 women reported pregnancy after MS diagnosis. Therefore, the lack of future reproductive plans should not be interpreted only as a consequence of MS-related concerns. In many cases, it may reflect completed family planning, age-related factors, or personal decisions unrelated to MS. At the same time, the relatively low number of pregnancies after MS diagnosis suggests that MS-related concerns may influence reproductive decisions in at least some patients. Previous studies also showed that women with MS may have lower fertility or live birth rates than the general population and that concerns related to MS may affect pregnancy planning [6,7,8,11]. These concerns may include fear of pregnancy, postpartum relapse, disability progression, treatment discontinuation, hereditary risk, difficulties in fulfilling parental responsibilities, and sexual dysfunction, which is more frequent in MS and may affect reproductive decisions and conception [1,15].

Current evidence indicates that MS itself does not usually worsen pregnancy outcomes or substantially increase obstetric risk [9,10]. Pregnancy is generally associated with reduced relapse activity, whereas relapse risk may increase in the postpartum period, particularly in patients with active disease or after treatment discontinuation [9,10,16]. In addition, pregnancy does not appear to have a significant adverse long-term effect on disability progression [11,16]. In our study, knowledge concerning the effect of pregnancy and the postpartum period on MS was limited, especially among men. This finding indicates that patient education should include not only the safety of pregnancy itself but also the expected changes in disease activity during pregnancy and after delivery.

The highest proportions of correct answers were observed for questions concerning disease-modifying therapy before conception and during pregnancy. For questions 6 and 8, both “Yes” and “It depends on the medicine used” were considered correct, reflecting the current individualized approach to treatment decisions. This point is important because recommendations regarding DMT use before conception, during pregnancy, and during breastfeeding have changed substantially in recent years [1,17]. Some therapies, such as interferon beta and glatiramer acetate, may be considered relatively safe in selected situations, whereas other drugs require specific precautions or discontinuation [1,17]. Therefore, patients should not assume that treatment or breastfeeding can always be continued without individual medical assessment. These decisions should be discussed with an MS specialist and should take into account the specific DMT, disease activity, relapse risk, and reproductive plans [1,17,18].

A low percentage of correct answers concerning treatment-related questions should not be interpreted only as a simple lack of patient knowledge. This area of MS management is complex and continues to evolve. In addition, discrepancies may exist between the Summary of Product Characteristics of individual drugs and expert recommendations based on emerging clinical evidence. This is particularly visible in relation to anti-CD20 therapies, for which recent expert recommendations may be less conservative than some product-label recommendations [19]. Therefore, uncertainty among patients may partly reflect the complexity and changing nature of this field. From a practical educational perspective, patients should not be expected to know detailed drug-specific algorithms. However, they should know that treatment decisions related to pregnancy and breastfeeding require individualized counseling by an MS specialist.

The lowest proportions of correct answers were observed for the question concerning breastfeeding and the course of MS, and for the question concerning treatment discontinuation in men before trying to have a child. Breastfeeding in MS remains a clinically relevant issue because it must be considered together with postpartum relapse risk and the timing of DMT resumption [1,19,20]. These findings suggest that breastfeeding and male reproductive issues are areas that require particular attention in patient education. Men had lower knowledge scores than women in almost all analyzed areas. This may reflect lower exposure to counseling about pregnancy and parenthood, as reproductive counseling in MS is often focused mainly on women. However, reproductive decisions may also involve male patients with MS, and counseling should address their information needs as well.

The present findings also allow comparison of women’s knowledge with the previous Polish study conducted in 2015 using a similar questionnaire [13]. Despite major advances in MS treatment and increasing availability of information, the level of knowledge among women did not appear to clearly improve over the last decade. This suggests that medical progress does not automatically translate into patient awareness. This interpretation is consistent with recent international data showing insufficient awareness of reproductive health issues among persons with MS [12]. Educational strategies should therefore be more structured, proactive, and repeated during routine MS care.

The insufficient level of knowledge observed in this study may result from several factors, and no single explanation can be identified. Some patients may have already completed family planning or may not consider pregnancy-related information relevant to their current life situation. Others may avoid the topic because of concerns about disability, treatment, or future parental responsibilities. General demographic trends, including declining birth rates, may also reduce interest in parenthood independently of MS [21]. Interestingly, many patients who searched for information considered it easy to obtain. This suggests that insufficient knowledge cannot be explained only by limited access to information. Another possible factor is insufficient discussion of pregnancy, parenthood, breastfeeding, and treatment-related issues during medical visits. The role of counseling is particularly important because neurologists and other healthcare professionals may differ in their approach to treatment decisions in women with MS who wish to become pregnant [22].

Greater efforts are needed to improve education for patients with MS, especially those of reproductive age. Discussions about pregnancy, parenthood, breastfeeding, and treatment planning should be a routine part of MS care [1,6,18,20]. Patients should have access to counseling by physicians experienced in MS management, with coordinated neurological and gynecological care when pregnancy is planned or occurs. Educational activities in MS centers and reliable online resources prepared under specialist supervision may also help improve patient knowledge and support informed reproductive decisions [12].

## 5. Limitations of the Study

This study has several limitations. First, the questionnaire was a study-specific tool and was not formally validated. No formal pilot testing, internal consistency analysis, or reliability testing was performed. Therefore, the results should be interpreted as an assessment of selected areas of patient knowledge, rather than as the result of a validated psychometric instrument.

Second, some questionnaire items, especially those concerning disease-modifying therapy before conception, during pregnancy, and during breastfeeding, used simplified response options. These answers were adapted to the expected level of patient knowledge and to the recommendations available when the questionnaire was developed and later updated. Current MS management in pregnancy is individualized and depends on the specific DMT, disease activity, relapse risk, and reproductive plans. Therefore, the questionnaire assessed general patient awareness rather than specialist-level knowledge of treatment algorithms.

Third, the threshold of ≥50% correct answers was arbitrary and should not be interpreted as a validated clinical standard of sufficient knowledge. It was used as a pragmatic descriptive cut-off to allow comparison with the previous study and between subgroups. The age cut-off of 35 years was arbitrary and was not based on a specific guideline or validated threshold. Therefore, age-related comparisons should be interpreted as exploratory.

Fourth, this was a single-center study with voluntary participation, and the cohort may not fully represent the broader MS population. The relatively limited sample size and unequal subgroup distribution, especially the small number of male participants and women who became pregnant after MS diagnosis, limited robust multivariable and subgroup analyses. In addition, the questionnaire did not collect detailed reproductive history before and after MS diagnosis; therefore, analyses based on whether pregnancy or fatherhood occurred before or after diagnosis could not be performed.

## 6. Conclusions

In this single-center cohort of patients with MS, knowledge about the impact of MS on pregnancy, childbirth, breastfeeding, fertility, and parenthood was limited.

Women achieved higher knowledge scores than men. However, conclusions concerning male participants should be interpreted with caution because of the relatively small size of the male subgroup, especially among men aged ≤35 years. Younger participants, particularly women, achieved higher mean knowledge scores than older participants.

The results suggest that education about pregnancy, parenthood, breastfeeding, and treatment planning should be improved in patients with MS. Counseling should be proactive, repeated during routine MS care, and adapted to both women and men. Future education should also specifically address the knowledge gaps observed among male patients with MS.

## Figures and Tables

**Table 1:** Demographic and clinical characteristics of patients with multiple sclerosis.

		Women *N* = 144 (74.2%)	Men *N* = 50 (25.8%)	Total *N* = 194
Age (years)	average (SD)	40.3 (10.3)	40.2 (10.5)	40.3 (10.3)
Education *	primary vocational secondary higher	4 (2.8%) 9 (6.2%) 36 (25%) 95 (66%)	2 (4%) 5 (10%) 13 (26%) 30 (60%)	6 (3.1%) 14 (7.2%) 49 (25.3%) 125 (64.4%)
Place of residence	village city <100,000 inhabitants city >100,000 inhabitants	19 (13.2%) 50 (34.7%) 75 (52.1%)	8 (16%) 15 (30%) 27 (54%)	27 (13.9%) 65 (33.5%) 102 (52.6%)
Monthly income, PLN	<3000 3000–5000 5000–8000 8000–10,000 >10,000 no answer	25 (17.4%) 36 (25.0%) 28 (19.4%) 14 (9.7%) 7 (4.9%) 34 (23.6%)	8 (16.0%) 13 (26.0%) 11 (22.0%) 5 (10.0%) 6 (12.0%) 7 (14.0%)	33 (17.0%) 49 (25.3%) 39 (20.1%) 19 (9.8%) 13 (6.7%) 41 (21.1%)
Relationship status	single in a relationship	35 (24.3%) 109 (75.7%)	16 (32.0%) 34 (68.0%)	51 (26.3%) 143 (73.7%)
Type of MS	relapsing-remitting secondary progressive primary progressive	128 (88.9%) 8 (5.6%) 8 (5.6%)	40 (80.0%) 4 (8.0%) 6 (12.0%)	168 (86.6%) 12 (6.2%) 14 (7.2%)
Age at diagnosis of MS (years)	average (SD)	31.5 (9.2)	30.2 (8.2)	31.2 (8.9)
Duration of disease (years)	average (SD)	8.8 (6.9)	10.0 (7.5)	9.1 (6.9)
Disability (EDSS **)	average (SD)	1.8 (1.6)	2.3 (1.8)	1.9 (1.6)

* Educational categories reflect the Polish education system. “Primary” indicates completed elementary/basic education; “vocational” indicates completed vocational education; “secondary” indicates completed general or technical secondary education; and “higher” indicates completed university-level education. ** Disability was assessed using a simplified patient-reported scale adapted from the Expanded Disability Status Scale (EDSS) [14]. MS, multiple sclerosis.

**Table 2:** Parenthood status and future reproductive plans among patients with multiple sclerosis.

		Women *N* = 144	Men *N* = 50	Total *N* = 194
Do you have children?	No Yes	55 (38.2%) 89 (61.8%)	24 (48.0%) 26 (52.0%)	79 (40.7%) 115 (59.3%)
How many children do you have?	None One child Two children Three children	55 (38.2%) 46 (31.9%) 35 (24.3%) 8 (5.5%)	Not applicable due to lack of data	
Do you consider your children to be healthy?	No (they perceive their children as sick) Yes (they perceive their children as healthy) Not applicable due to not having children	7 (4.9%) 82 (56.9%) 55 (38.2%)	0 26 (52.0%) 24 (48.0%)	7 (3.6%) 108 (55.7%) 79 (40.7%)
Are you currently pregnant?	No Yes	143 (99.3%) 1 (0.7%)	Not applicable	
Were these pregnancies planned?	Yes, all of them No, some of them were not. No, none of them. Not applicable due to not being pregnant.	72 (50.0%) 13 (9.0%) 4 (2.8%) 55 (38.2%)	18 (36.0%) 1 (2.0%) 6 (12.0%) 25 (50.0%)	90 (46.4%) 14 (7.2%) 10 (5.1%) 80 (41.2%)
When trying to conceive, did you know that you had multiple sclerosis?	Yes No I did during diagnosis. Not applicable due to not being pregnant.	22 (15.3%) 75 (52.1%) 3 (2.1%) 44 (30.5%)	14 (28.0%) 14 (28.0%) 0 22 (44.0%)	36 (18.5%) 89 (45.9%) 3 (1.5%) 66 (34.0%)
Have you been pregnant after being diagnosed with MS?	No Yes	125 (86.8%) 19 (13.2%)	Not applicable	
Does multiple sclerosis influence your decision to have children?	Yes, it’s a key factor. Yes, but there are also other factors not related to MS. No, MS does not have an impact on my decision. I do not know.	22 (15.3%) 41 (28.5%) 66 (45.8%) 15 (10.4%)	10 (20.0%) 11 (22.0%) 21 (42.0%) 8 (16.0%)	32 (16.5%) 52 (26.8%) 87 (44.8%) 23 (11.8%)
Do you want to have children in the future, either your first child or another child?	Yes, as soon as possible. Yes, but not now. Probably not Definitely not I have not thought about it yet I am currently pregnant/My partner is currently pregnant.	8 (5.6%) 23 (16.1%) 39 (27.3%) 69 (48.2%) 3 (2.1%) 1 (0.7%)	8 (16.0%) 12 (24.0%) 13 (26.0%) 15 (30.0%) 0 2 (4.0%)	16 (8.3%) 35 (18.1%) 52 (26.9%) 84 (43.5%) 3 (1.5%) 3 (1.5%)

**Table 3:** Questionnaire items assessing knowledge about the impact of multiple sclerosis on pregnancy, childbirth, breastfeeding, and parenthood.

Question	Possible Answers
1. In your opinion, how does multiple sclerosis affect the course of pregnancy?	Rather unfavorable—it increases the risk of obstetric complications Rather favorable—enables better control of the baby’s development during pregnancy No influence I have no opinion on this subject.
2. In your opinion, how does pregnancy affect the course of multiple sclerosis?	Rather unfavorable—it increases the frequency and severity of relapses Rather favorable—it decreases the frequency and severity of relapses. No influence I have no opinion on this subject.
3. In your opinion, how does the postpartum period affect the course of multiple sclerosis?	Rather unfavorable—it increases the frequency and severity of relapses. Rather favorable—it decreases the frequency and severity of relapses. No influence I have no opinion on this subject.
4. In your opinion, how does breastfeeding affect the course of the disease?	Rather unfavorable—it increases the frequency and severity of relapses Rather favorable—it decreases the frequency and severity of relapses. No influence I have no opinion on this subject.
5. Do you think that a child has a higher risk of developing multiple sclerosis if one of the parents has the disease?	Yes, the risk is significantly increased. Yes, but it is only slightly increased. No, the risk is not increased. I do not know
6. In your opinion, should disease-modifying therapy be discontinued in women with multiple sclerosis before trying to conceive?	Yes No It depends on the medicine used. I do not know
7. In your opinion, should disease-modifying therapy be discontinued in men with multiple sclerosis before trying to father a child?	Yes No I do not know
8. In your opinion, should disease-modifying therapy be discontinued during pregnancy?	Yes No It depends on the medicine used. I do not know
In your opinion, should disease-modifying therapy be discontinued during breastfeeding?	Yes No I do not know
10. In your opinion, does multiple sclerosis reduce fertility?	Yes No I do not know
11. In your opinion, is cesarean section necessary in women with multiple sclerosis?	Yes, multiple sclerosis is an absolute indication for cesarean section. No, unless there are other indications, cesarean section is not necessary. I do not know
12. In your opinion, are newborns of women with multiple sclerosis at higher risk of low birth weight, prematurity, or poorer condition immediately after birth compared with newborns of women without MS?	Yes, definitely often. No, there is no significance. I do not know

**Table 4:** Mean number of correct answers in the knowledge test according to sex and age group.

	Age Groups		*p* Value *
	≤35 Years Old (*n* = 65) Average (SD)	>35 Years Old (*n* = 129) Average (SD)	
Total (*n* = 194)	5.0 (2.9)	3.2 (2.6)	<0.001
Women (*n* = 144)	5.9 (2.6)	3.3 (2.6)	<0.001
Men (*n* = 50)	2.7 (2.1)	2.8 (2.3)	0.984

* Mann–Whitney U test.

**Table 5:** Number and percentage of participants reaching the predefined threshold of ≥50% correct answers.

	Age Groups		*p* Value *
	≤35 Years Old (*n* = 65) *n/N* (%)	>35 Years Old (*n* = 129) *n/N* (%)	
Total	25/65 (38.5%)	36/129 (27.9%)	0.135
Women	23/47 (48.9%)	28/97 (28.9%)	0.018
Men	2/18 (11.1%)	8/32 (25.0%)	0.239

* Chi-squared test.

**Table 6:** Number and percentage of patients with multiple sclerosis giving correct answers to individual knowledge-test items.

	Total (*N* = 194)	Women (*N* = 144)	Men (*N* = 50)
Question 1	48 (24.7%)	38 (26.4%)	10 (20%)
Question 2	50 (25.8%)	43 (29.9%)	7 (14%)
Question 3	61 (31.4%)	52 (36.1%)	9 (18%)
Question 4	8 (4.1%)	6 (4.2%)	2 (4%)
Question 5	58 (29.9%)	46 (31.9%)	12 (24%)
Question 6	116 (59.8%)	92 (63.9%)	24 (48%)
Question 7	22 (11.3%)	18 (12.5%)	4 (8%)
Question 8	113 (58.2%)	94 (65.3%)	19 (38%)
Question 9	45 (23.2%)	40 (27.8%)	5 (10%)
Question 10	71 (36.6%)	53 (36.8%)	18 (36%)
Question 11	84 (43.3%)	67 (46.5%)	17 (34%)
Question 12	63 (32.5%)	52 (36.1%)	11 (22%)

**Table 7:** Percentage of patients with multiple sclerosis aged ≤ 35 years giving correct answers to individual knowledge-test items.

	Total (*N* = 65)	Women (*N* = 47)	Men (*N* = 18)
Question 1	30.8%	34%	22.2%
Question 2	40%	48.9%	16.7%
Question 3	43.1%	55.3%	11.1%
Question 4	4.6%	6.4%	0%
Question 5	40%	46.8%	22.2%
Question 6	84.6%	91.5%	66.7%
Question 7	12.3%	14.9%	5.6%
Question 8	72.3%	83%	44.4%
Question 9	27.7%	34%	11.1%
Question 10	43.1%	51.1%	22.2%
Question 11	61.5%	74.5%	27.8%
Question 12	40%	48.9%	16.7%

**Table 8:** Information-seeking about multiple sclerosis, pregnancy, and childbirth and sources of information among patients with multiple sclerosis.

Question	Women *N* = 144	Men *N* = 50	Total *N* = 194
Have you ever searched for information about the relationship between multiple sclerosis, pregnancy, and childbirth? (only YES answers)	44 (30.5%)	7 (14.0%)	51 (26.3%)
If “YES”, what was your source?			
neurologist	31 (21.5%)	2 (4.0%)	33 (17.0%)
gynecologist	17 (11.8%)	0	17 (8.8%)
midwife	3 (2.1%)	0	3 (1.5%)
websites	29 (20.1%)	4 (8.0%)	33 (17.0%)
internet forums	9 (6.2%)	1 (2.0%)	10 (5.1%)
Patient leaflets or educational brochures	16 (11.1%)	1 (2.0%)	17 (8.8%)
Other	3 (2.1%)	3 (2.1%)	4 (2.1%)

## Data Availability

The data presented in this study are available upon request from the corresponding author.

## Abbreviations

The following abbreviations are used in this manuscript:

MS	multiple sclerosis
